# Editorial: Behavioral and Neural Bases of Object Affordance Processing and Its Clinical Implications

**DOI:** 10.3389/fnhum.2021.759377

**Published:** 2021-09-24

**Authors:** Sanjay Kumar, Patric Bach, Dimitrios Kourtis

**Affiliations:** ^1^Psychology, Oxford Brookes University, Oxford, United Kingdom; ^2^School of Psychology, University of Aberdeen, Aberdeen, United Kingdom; ^3^Psychology, University of Stirling, Stirling, United Kingdom

**Keywords:** affordance, attention, action, EEG, compatibility

Gibson's, 1979 concept of affordances had a major impact on psychology and neuroscience. The term denotes the actions that the environment makes possible—“affords”—for the organism in a situation: that this chair may be sat on, that this ball could be thrown about this far, and that this vase might break if not handled carefully. In Gibson's original formulation organisms would “directly perceive” those affordances that are relevant to them, given their current action capabilities, allowing them to carry out those most in their line with their goals.

Research since then has shown that people are indeed exquisitely sensitive to the affordances around them, in a manner that is precisely calibrated to their body's real capabilities. For example, whether people perceive stairs as climbable can be predicted from the ratio of stair height to their leg length (Warren, 1984), and similar scaling have been demonstrated for other actions too, such as reaching and grasping (Cesari and Newell, 1999), taking into account any tools one may have access to (Farnè and Làdavas, 2000). People even make these judgments for others (Stoffregen et al., 1999), suggesting that actors do not only understand what the environment affords *them* in a situation, but also the people around them (Bach et al., 2014; McDonough et al., 2020).

Research in psychology and neuroscience has focussed on identifying the cognitive and brain mechanisms that achieve this translation of organism-relevant environmental features into action. In a seminal series of experiments, Tucker and Ellis (1998, 2001) demonstrated that simply seeing an object biases behaviour toward actions that match the object features (e.g., precision grips for small objects, power grips for large objects; see also Tipper et al., 2006; Bub and Masson, 2010). Neuroimaging brought complementary evidence that actionable objects activate respective part of the neuronal system involved in controlling the relevant actions toward them (Grezes and Decety, 2002; Roux-Sibilon et al., 2018). Neuropsychology showed that the affordances of an object, and how they fit current action plans, directly impact how the world is perceived (Humphreys and Riddoch, 2001; Riddoch et al., 2003).

These findings demonstrated that affordances have a neuro-cognitive reality and play a substantial role in structuring behaviour (for reviews, see Buxbaum and Kalénine, 2010; Thill et al., 2013; Borghi and Riggio, 2015). Yet, their precise role in theory, action control and cognition are hotly debated. For example, it is unclear (a) whether affordance perception depends on attention, or whether, conversely, affordances guide attention to goal-relevant objects (Humphreys et al., 2013). It is debated (b) whether affordances are perceived dynamically, relative to the current environmental context and capabilities and goals of the actor, or whether they are retrieved in a more rigid manner, based on memory of prior interactions with the object (Bub et al., 2008; Kourtis and Vingerhoets, 2015; Osiurak et al., 2017; Kourtis et al., 2018). Research has (c) also asked how affordance processing relates to space, both in terms of a common spatial coding of object and body features (Cho and Proctor, 2013), and in terms of how affordances and our own action capabilities structure the space we act in, individually and also with others (Farnè and Làdavas, 2000).

This special issue was convened to bring together top researchers in the field of affordances and provide a forum to discuss and potentially resolve some of these open questions. The contributions we received provide a microcosm of the above issues and provide a spotlight on current research that attempts to resolve them.

Scerrati et al. test whether the functional properties of objects—what they are *for* and whether they are configured to achieve this function—play a role in facilitating action, in other words, whether affordances drive action especially for objects with which goals can potentially be achieved. Their research therefore links affordance perception to prospective control and how it helps the organism achieve their higher-level goals (Turvey, 1992).

Osiurak et al. challenge the prevalent view that the perception of affordances, and their translation into action, is governed by memory of rigid object-behaviour links (e.g., that perception of a hammer invokes the representation of hammering). Instead, their theoretical account supports the notion of a technical reasoning process that makes use of the currently available transformations in the environment and combines them to make a variety of complex actions possible.

Costantini et al. test the assumption that affordance perception and action are deeply intertwined. They ask whether the link between objects and current actions already bias perception and attention toward those objects one can most effectively interact with. They indeed show that people identify even non-affording object features (such as colour) more quickly when the object itself is linked to one's current actions.

Keric and Sebanz investigate the idea that affordance perception is tuned to the current capabilities of the body. They put to test the influential view that the effort when interacting with object biases perception of their visuospatial attributes (e.g., that the perceived height of an object depends on how effortfully it could be reached). Their results invite scepticism for this idea, at least for the way this is typically tested and suggest that effort-based effects in the prior literature may reflect how people construe the tasks rather than a feature of perceptual processing itself.

Kumar et al. investigate the processes that link the properties of an object to relevant behaviour. They reveal that a component in the EEG, the N2pc, indicative of visual selection, is sensitive to the match of a hand posture and the type of action an object affords. Their results therefore provide evidence that the matching of objects to the body determines attentional selection.

Finally, Coello and Cartaud provide a theoretical account of the role of peripersonal space in structuring one's interaction with both objects and other people, guiding actions toward rewarding interactions and away from those that are potentially harmful. They advance the proposal that peripersonal space may flexibly guide interactions with objects and other people, and act as a buffer against unwanted interactions.

## Author Contributions

All the authors contributed to the draft and approved the final submission.

## Conflict of Interest

The authors declare that the research was conducted in the absence of any commercial or financial relationships that could be construed as a potential conflict of interest.

## Publisher's Note

All claims expressed in this article are solely those of the authors and do not necessarily represent those of their affiliated organizations, or those of the publisher, the editors and the reviewers. Any product that may be evaluated in this article, or claim that may be made by its manufacturer, is not guaranteed or endorsed by the publisher.
